# No fitness effects of same‐sex copulations in male red flour beetles

**DOI:** 10.1002/ece3.11027

**Published:** 2024-03-19

**Authors:** Birte M. Martens, Oliver Y. Martin, Tim Janicke, Lennart Winkler

**Affiliations:** ^1^ Applied Zoology TU Dresden Dresden Germany; ^2^ Department of Biology & Institute of Integrative Biology (IBZ) ETH Zürich Zurich Switzerland; ^3^ Centre d'Écologie Fonctionnelle et Évolutive, UMR 5175, CNRS Université de Montpellier, École Pratique des Hautes Études Montpellier Cedex 05 France

**Keywords:** same‐sex sexual behavior, sexual indiscrimination, sperm competition, sperm dumping, sperm translocation, *Tribolium castaneum*

## Abstract

Same‐sex sexual behavior occurs in diverse animal taxa, yet its evolutionary maintenance is poorly understood as such behavior seems to be costly and does not directly increase reproductive success. We used male *Tribolium castaneum* beetles, which frequently engage in same‐sex copulations, to test if same‐sex sexual behavior influences future male mating behavior and reproductive success of males. Furthermore, we tested whether same‐sex sexual behavior has benefits via indirect sperm translocation. We conducted a series of mating trials demonstrating that males exposed to same‐sex behavior did not sire less offspring compared to control males that did not engage in same‐sex behavior. This suggests that same‐sex copulations did not lead to fitness costs in subsequent mating interactions. In addition, we found no evidence that indirect sperm translocation via an intermediate male occurs in *T. castaneum*. Taken together, these results imply that same‐sex sexual behavior in males is associated with no costs in terms of lower mating rate and reduced siring success and does not seem to entail benefits. Moreover, our data conform to the hypothesis that sexual indiscrimination is prevalent in this species, which may explain the relatively high frequency of same‐sex sexual behavior in *T. castaneum*.

## Graphical abstract

We find that same‐sex matings have limited costs in the red‐flour beetle, which may relax selection for efficient sex recognition. Same‐sex copulations are an evolutionary paradox because they do not result in offspring and hence seem a waste of resources. We examined the mating behavior and future reproductive success of male red flour beetles performing same‐sex behavior.

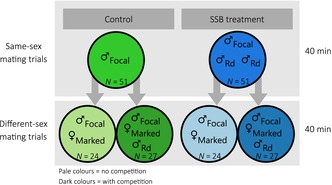

## 1 INTRODUCTION

Same‐sex sexual behavior (SSB) has been found to occur in many vertebrate and invertebrate taxa (Bailey & Zuk, 2009; Monk et al., 2019; Scharf & Martin, 2013). Furthermore, it may exist in many more species for which it has not been observed yet or has been misidentified as different‐sex sexual behavior (DSB) due to a lack of obvious sexual dimorphism (Bailey & Zuk, 2009). SSB is often viewed as an evolutionary paradox since it is assumed to have no reproductive benefits. Instead, SSB might have costs because individuals engaging in SSB spend time, energy, and potentially resources that they could instead invest into DSB. Therefore, research has often sought to explain the adaptive significance of SSB by searching for benefits of SSB that outweigh the costs (Bailey & Zuk, 2009; Monk et al., 2019; Scharf & Martin, 2013).

Overall, our knowledge concerning the net effect of SSB on reproductive fitness is very limited (Bailey & Zuk, 2009; Dukas, 2010; Maklakov & Bonduriansky, 2009; Monk et al., 2019). Potential benefits of SSB include the gain of social dominance, conflict avoidance, and increased sexual experience, and recent work suggests that costs may be smaller than often assumed, particularly in species with high‐mating frequencies or species that invest little into individual mating attempts (Hoving et al., 2011; Lerch & Servedio, 2021; Monk et al., 2019; Scharf & Martin, 2013). In fact, sexual indiscrimination might be an ancestral trait (Monk et al., 2019), though it does not explain its current prevalence in many species (Dickins & Rahman, 2020). Theoretical work suggests that being sexually indiscriminate and engaging in both SSB and DSB can be an evolutionary stable strategy for the searching sex under a wide range of conditions (Lerch & Servedio, 2021). Furthermore, exclusive DSB requires mechanisms of sex recognition, which could only have developed after the evolution of perceivable sexual dimorphism (Monk et al., 2019). Being sexually indiscriminate and engaging in both DSB and SSB could decrease the chance of falsely rejecting a possible mate (Richardson & Zuk, 2023). In addition, high discrimination could involve costs in the development and maintenance of behavioral and morphological secondary sexual traits and the detection of these traits (Monk et al., 2019; Sales et al., 2018; Serrano et al., 1991; Thornhill & Alcock, 1983).

Here, we aim to expand our limited knowledge of the adaptive significance of SSB by investigating its effect on mating behavior and reproductive success using the red flour beetle *Tribolium castaneum* as a model system. *T. castaneum* is known to mate frequently (Wool, 1967) and with multiple partners (Lewis & Iannini, 1995; Martin et al., 2015; Winkler et al., 2023). Males display sexual behavior with both females and males (Graur & Wool, 1982; Rich, 1972, 1989; Sales et al., 2018; Wool, 1967) with observed rates of SSB ranging from 11.4% (Graur & Wool, 1982) to 44.6% (Rich, 1972). Nevertheless, it is unknown how common SSB is within their flour habitat since beetles mate inside of tunnels (Fedina & Lewis, 2008). Male SSB in *T. castaneum* closely resembles the behavior involved in different‐sex copulations (LeVan et al., 2009; Martin et al., 2015). It consists of a male climbing on another male's back, with both beetles facing the same direction, extending its aedeagus, and engaging in leg‐rubbing behavior (LeVan et al., 2009). Furthermore, it often involves the release of a spermatophore by the mounting male (LeVan et al., 2009; Spratt, 1980). Interestingly, SSB has been shown to have a genetic component in *T. castaneum* suggesting consistent individual differences and the potential that this behavior can evolve (Castro et al., 1994; Serrano et al., 1991).

Previous work suggests that SSB is costly in *T. castaneum*. In particular, males kept in same‐sex groups have significantly shorter lifespans than males kept in mixed groups or in isolation (Spratt, 1980), indicating that there are costs of SSB. Furthermore, there seem to be costs of SSB in terms of lower mating rates in subsequent male–female copulations for males kept in same‐sex groups (LeVan et al., 2009). Yet, rearing males in same‐sex groups may affect many more variables, such as general stress levels, and it is difficult to disentangle these from the effect of SSB. Other potential costs of SSB include resources and time that individuals invest during SSB without direct fitness returns. This can also negatively affect an individual's future chances for successful reproduction, as, for example, frequent SSB could lead to sperm depletion. Sperm depletion through SSB could be particularly detrimental to male fitness under sperm competition (Smith et al., 2009). Furthermore, it remains unknown how SSB influences mating behavior in the short term, which seems a more likely ecological scenario in mixed‐sex groups. Most importantly, the net effect of SSB on male reproductive success remains untested.

Earlier studies on SSB in *T. castaneum* could not identify benefits that would explain the maintenance of this behavior (LeVan et al., 2009; Sales et al., 2018). SSB does not appear to be a mechanism to gain dominance (LeVan et al., 2009; Sales et al., 2018) or to practice for future heterosexual encounters (LeVan et al., 2009). LeVan et al. (2009) proposed that SSB might be a mechanism of indirect sperm transfer through an intermediary male similar to observed DSB interactions. Specifically, Haubruge et al. (1999) studied DSB and found that indirect sperm transfer of males via an intermediate, previously mated female occurred often in consecutive crosses, with 22% of females having offspring that was not sired by their actual mating partner. They suggested that chitinous spines on the male's aedeagus removed sperm of previous mates from the female's *bursa copulatrix*. Their study was later replicated using a different paternity marker but no evidence of indirect sperm transfer could be found (Tigreros et al., 2009). LeVan et al. (2009) suggested that a similar mechanism could occur during SSB. Specifically, males might transfer sperm to the partner's male genitalia when performing SSB, which the partner subsequently may transfer to a female during mating. Still, there was only limited evidence for this, with only 0.5% of each female's total progeny where indirect sperm transfer occurred being the product of indirect sperm transfer (LeVan et al., 2009). It is hence questionable whether this effect is widespread and strong enough to explain the occurrence of SSB in *Tribolium*.

In many species, sperm quality declines with age (Reinhardt, 2007) and male *T. castaneum* might use SSB to discard old sperm, to make room for better quality, younger sperm (LeVan et al., 2009). *T. castaneum* males do not only engage in sexual behavior with other males but also with dead beetles and even objects that loosely resemble beetles (Taylor & Sokoloff, 1971). This could be a mechanism that helps males to discard old sperm but might alternatively also be the result of indiscriminate mating by males (Richardson & Zuk, 2023). However, the transfer of low‐quality sperm to females is likely to be advantageous for males in polygamous species with no post‐zygotic paternal investment. This is because mating with a female should convey higher fitness compared to SSB as the likelihood of successful fertilization is still non‐zero even if poor‐quality ejaculates usually show decreased sperm competitiveness (Michalczyk et al., 2010). Following the hypothesis that SSB serves as a mechanism to discard old sperm, one might expect males to only engage in SSB when no females are present, to ensure that fresh sperm is available if a female appears. In support of this hypothesis, LeVan et al. (2009) found that isolated males show more SSB. However, a higher frequency of SSB by isolated males may also reflect a higher mating propensity of sex‐deprived males (Taylor & Sokoloff, 1971; Wool, 1967), making it difficult to discern whether isolation influenced SSB or the overall eagerness to mate.

The relatively high frequency of SSB in *Tribolium* may result from imperfect sex recognition. *T. castaneum* males appear to be sexually indiscriminate, meaning that they do not or cannot discriminate between males and females (Sales et al., 2018; Serrano et al., 1991, 2000; Taylor & Sokoloff, 1971). In fact, there is no external morphological dimorphism between males and females, beyond the subtle difference that males have setiferous glands located on the femurs of their prothoracic legs (Faustini et al., 1981; LeVan et al., 2009; Serrano et al., 1991) and that females typically have a greater body mass compared to males (Winkler & Janicke, 2022). Their highly similar appearance combined with living and mating within a substrate makes visual perception and communication unlikely. There is also no evidence for acoustic communication, as *Tribolium* beetles have no apparent specialized tympanal organs (Serrano et al., 1991). Olfaction may be the main way of communication in *T. castaneum*, as males secrete an aggregation pheromone that attracts both sexes (Boake & Wade, 1984; Faustini et al., 1981; Levinson & Mori, 1983; Serrano et al., 1991). Nevertheless, it remains largely unknown how olfactory cues might assist sex recognition in *T. castaneum*, but inter‐strain variation in SSB might be linked to olfactory cues (Rich, 1989). For these reasons, discrimination between males and females may be difficult and costly for males who are searching for a sexual partner. Observed rates of SSB fit a model that assumes total indiscrimination by males (Serrano et al., 1991, 2000). Nevertheless, Sales et al. (2018) found that males in strongly male‐biased populations were selected for better ability to discriminate between the sexes, whereas males from female‐biased populations mated seemingly at random. It is possible that the cost of engaging in SSB is balanced by or lower than the costs of potentially losing different‐sex mating opportunities (Monk et al., 2019; Richardson & Zuk, 2023; Sales et al., 2018). This would favor the evolution of a broad filter for mating decisions, where males tend to mate with everything that resembles a potential partner (Richardson & Zuk, 2023). Cryptic female choice in terms of accepting or rejecting a male's spermatophore (Bloch Qazi, 2003; Fedina, 2007; Fedina & Lewis, 2006, 2007, 2008) and last‐male sperm precedence (Arnaud & Haubruge, 1999; Edvardsson & Arnqvist, 2000, 2005; Fedina & Lewis, 2006; Graur & Wool, 1982; Haubruge et al., 1999) may favor high mating rates in males.

Here we investigated the effect of SSB on mating behavior and its fitness consequences in males. We observed the mating behavior of males in two consecutive trials comprising a same‐sex mating trial followed by a male–female mating trial. On the assumption that SSB is not the result of males being sexually indiscriminate, we expect that:

H1. SSB modulates male mating behavior. We compared the number, duration, and latency of male–female copulations between males that engaged in SSB and control males that did not engage in SSB. We predicted that males involved in SSB are less prone to invest in subsequent mating behavior, which should manifest in a lower number of copulations, shorter average copulation duration, and an increased copulation latency.
H2. SSB impacts male reproductive success. We predicted that males that performed SSB sire less offspring. In addition, the frequency of SSB performed before a male–female mating should predict the reproductive success of males.
H3. SSB impairs male competitiveness in terms of lower paternity share as a consequence of sperm depletion. We expected that males who performed SSB have a lower paternity share compared to control males who did not engage in SSB.
H4. Replicating previous ambiguous findings (Haubruge et al., 1999; LeVan et al., 2009; Tigreros et al., 2009), we hypothesize that SSB functions to indirectly transfer sperm to females via an intermediate male. If indirect sperm transfer occurs in *T. castaneum*, we predict that females would produce offspring that are sired by males they did not mate with. This would be the result of sperm transferred via an intermediate male that engaged in SSB with the sire prior to mating with the female.

## 2 METHODS

### 2.1 Study animals

All focal males and female mating partners were from laboratory stock cultures of the wildtype strain Georgia 1 (GA1) of *T. castaneum* supplied by the U.S. Department of Agriculture and kept in our laboratory for over a year. Stock cultures as well as all experimental cultures were kept in a flour mixture that consisted of organic wheat flour (*Alnatura* type 405, Darmstadt, Germany) supplemented with 5% dry baker's yeast. All culture vials were stored in a dark incubator at 30°C and 40%–50% humidity. Same‐sex mating partners and competitor males were “Reindeer honey dipper” (hereafter Rd). Rd is a dominant mutation that affects antenna morphology (i.e., Rd has a thicker and shorter antenna compared to the wildtype), making the Rd mutant easily distinguishable from the wildtype, and useful as a paternity marker. The Rd stock cultures were kept under the same conditions in the laboratory as the wild‐type stain.

Pupae of similar age (±2 days at egg laying) were separated by sex and distributed over the treatments. Females were kept in large, single‐sex groups of 100 individuals in 50 g of flour mixture and were marked with *Revell* enamel paint (Revell GMBH, Bünde, Germany) at least 1 day before being used in the mating trials. All 102 wild‐type males and 285 Rd males were kept isolated in 1 g of flour mixture in 20 mL scintillation vials. All individuals were fully adult when starting the experiment (GA1 12–14 days and Rd 8–14 days from eclosion). Mating trials were performed consecutively over 2 weeks.

### 2.2 Same‐sex mating trials

First, we performed same‐sex mating trials, which lasted 40 min and consisted of a focal male being placed with two Rd males in an observation arena. The arenas were plastic vials (diameter: 2.2 cm, height: 0.9 cm) that were scratched at the bottom to allow better traction. Arenas were thoroughly washed with tap water before reuse in subsequent trials. We performed 51 same‐sex mating trials (hereafter “SSB treatment”). In addition, we set up 51 control trials following the same procedure as for the SSB treatment, but not adding any other males to the arena. Hence, males in the control group could not perform SSB in these trials. All behavioral interactions were recorded inside an incubator using a *Sony HDR‐CX240EB* video camera, enabling up to six trials at the same time. For each replicate, we recorded the number of copulations initiated by the focal male, the number of times the focal male was mounted by a Rd male, copulation duration (in seconds), and copulation latency (the time from the beginning of the recording to the first copulation by the focal male in seconds). Throughout, we defined copulations as a behavior in which the male climbed onto the back of another beetle (in this case a male) with his body in the same orientation, extending the aedeagus towards the mounted partner for at least 29 s (see Section 2.4 for details). The Rd males were discarded after the trials and the focal males were left in the arenas to participate in the male–female mating trials that occurred immediately after.

### 2.3 Different‐sex mating trials and fitness assays

**FIGURE 1:**
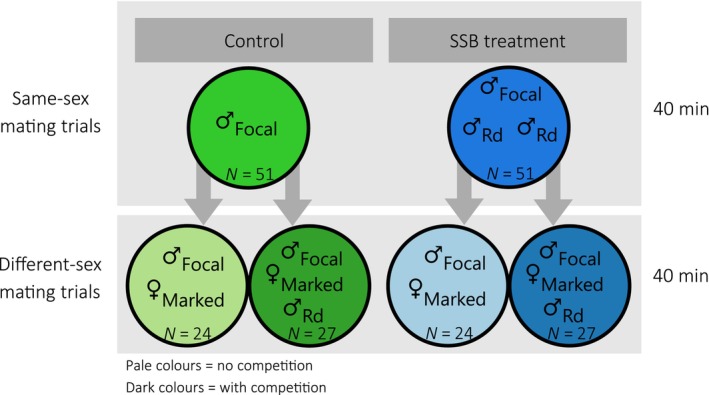
Experimental scheme to investigate the effect of same‐sex sexual behavior on male reproduction in *Tribolium castaneum*.

Second, we carried out male–female mating trials, which lasted 40 min and consisted of one wildtype female being placed into the observation arena with the focal male. A total of 102 focal males were observed (51 SSB‐treated males and 51 control males). A male Rd competitor was added to the male–female mating trials in every second trial. Thus, in 54 of the male–female mating trials (27 of SSB‐treated males and 27 of control males), focal males faced pre‐ and postcopulatory sexual selection imposed by the presence of an Rd competitor male, enabling us to observe the fitness consequences of SSB under male–male competition (e.g. to detect potential effects of sperm depletion). Furthermore, competition could affect mating behavior, for example by reducing mating duration due to interference by the competitor. As multiple mating is the norm in *T. castaneum* (Lewis & Iannini, 1995; Martin et al., 2015; Winkler et al., 2023), this treatment more closely resembled natural conditions compared to the treatment without competition. We recorded the number and type (same‐sex or male–female) of copulations (as defined above) initiated by the focal male, copulation latency (time to start copulations), and duration of copulations. Males were discarded after the trials and females were placed singly into vials with 8 g flour mixture, where they were left to oviposit. After 1 week, females were transferred to another vial and left to oviposit for another week. The females were removed and discarded after these 2 weeks. The number and type (i.e., wildtype or Rd) of offspring was determined 6 weeks after the females had been removed, once all offspring were adult. Behavioral analyses and offspring counting were performed blindly with regard to the treatment (Figure 1).

### 2.4 Statistical analysis

Statistical analysis was carried out using R (V4.3.1; R Core Team, 2023). We used general linear models (GLMs) of the quasi‐Poisson family for count data and quasibinomial GLMs for proportional offspring data because data were not normally distributed and were overdispersed. GLMs for copulation duration were of the Gaussian family. For testing the overall effect of the treatment, we report ANOVA results. For behavioral data, we ran models including both the SSB treatment (i.e., “SSB” or “control”) and competition treatment (i.e., “no competition” or “with competition”), as well as their interaction. When the interaction was significant (*p* < .05), we used type III ANOVAs and when the interaction was non‐significant we used type II ANOVAs. Finally, we tested if indirect sperm translocation had occurred by counting the number of Rd offspring in females that had not copulated with an Rd male (i.e., females from the treatment without competition). We compared this number with the result of a previous study (LeVan et al., 2009) using a Chi‐squared test.

We used a threshold of 29 s for mountings to be included in our analyses as successful copulations. This threshold was based on the observation that previously unmated females produced offspring after being mounted for at least 29 s, indicating that sperm transfer occurred within that time in the present study. Mounting events of a duration longer than 40 s have, however, been used as thresholds in previous studies (Attia & Tregenza, 2004; Bloch Qazi et al., 1996). An alternative analysis using a threshold of 39 s mounting duration (after Bloch Qazi, 2003) showed qualitatively similar results (see Appendix 1: Tables A1, A2, A3, A4) to the ones reported below. We excluded data from same‐sex trials where we did not observe SSB of the focal from all analyses (*N* = 4), as these males did not differ in SSB history from the control.

## 3 RESULTS

Overall, we observed that 92.16% of focal *T. castaneum* males initiated copulations in same‐sex mating trials and 95.15% of focal males copulated with the female in different‐sex mating trials. The duration of same‐sex mountings varied from less than 5 s to 9.08 min. Males subjected to the same‐sex treatment mounted in 47 of 51 trials at least one of the Rd males, with a median of 3 and a maximum of 11 same‐sex copulations by focal males. The mean latency for same‐sex copulations was 326 s, with a mean duration of 45 s.

### 3.1 Higher copulation latency of males exposed to same‐sex behavior

**FIGURE 2:**
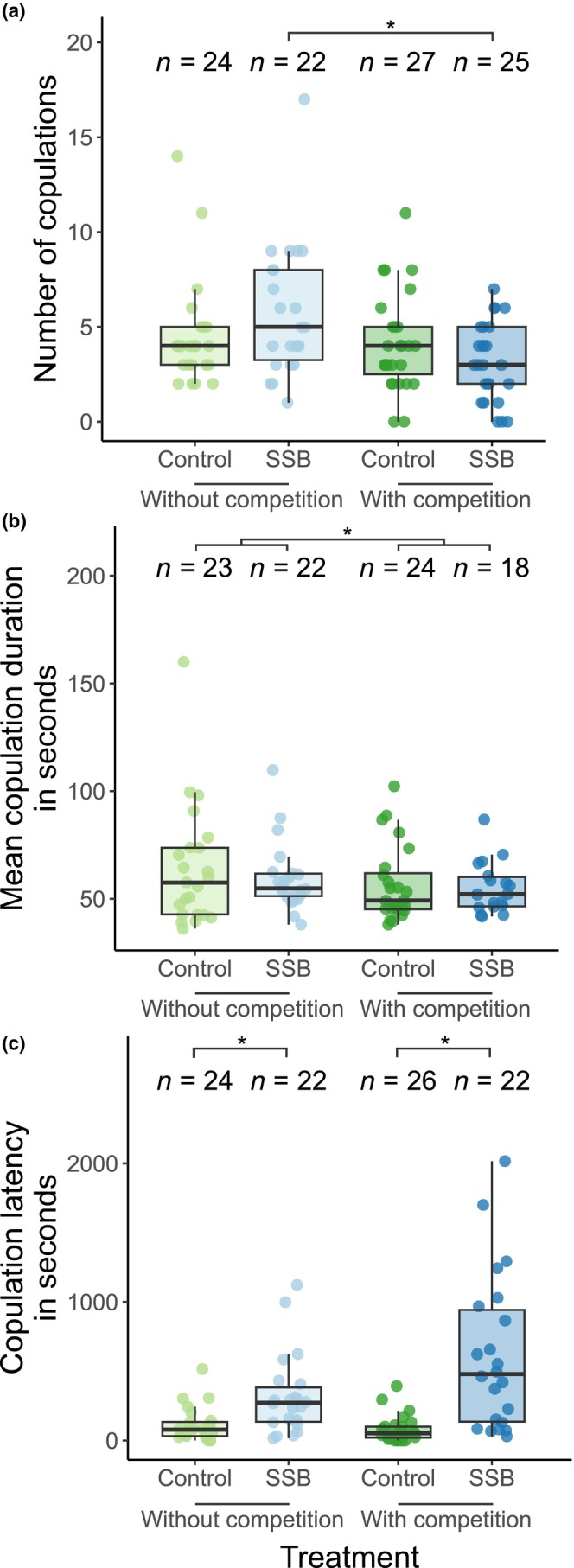
Comparison of mating behavior of males subjected to same‐sex mating trials (blue) and control males that did not engage in SSB (green). Mating trials without competition (pale colors) and with competition (dark colors). (a) Number of copulations initiated by males, (b) mean duration of the copulations initiated by males, (c) copulation latency (duration to first mounting by males). Boxplots show median, first/third quantiles and whiskers indicate data range excluding outliers. Bars with an asterisk indicate significant treatment effects (see Table 1).

First, we examined if exposure to SSB affected the mating behavior of males in subsequent matings with females. In different‐sex mating trials, focal males faced either no competition or a Rd competitor male. There was no difference in the number or the duration of copulations between SSB‐exposed and control males (Figure 2a,b, Table 1). By contrast, copulation latency was significantly longer in males from the same‐sex treatment compared to control males (Figure 2c, Table 1). Copulations were significantly shorter in the presence of a competitor (Figure 2b, Table 1). However, there was no significant interaction between the SSB treatment and the competition treatment (Table 1). By contrast, the number of copulations was only reduced by competition in the SSB‐treated males but not in control males, as indicated by a significant interaction between the treatments (Figure 2a, Table 1). Likewise, copulation latency was only increased by the competition treatment in SSB‐treated males and not in control males (Figure 2c, Table 1).

**TABLE 1:** Effect of same‐sex sexual behavior treatment and competition treatment on the mating behavior of males during different‐sex mating trials.

Trait	Model term	Estimate	SE	*χ* ^2^	*p*
Number of copulations df = 94 *R* ^2^ = .11	SSB treatment	0.26	0.17	2.29	.130
	Competition treatment	−0.09	0.18	0.26	.608
	SSB × Competition	−0.52	0.26	4.15	.042
Mean duration df = 89 *R* ^2^ = .07	SSB treatment	−15.22	9.77	2.07	.150
	Competition treatment	−19.37	9.46	4.39	.036
	SSB × Competition	10.53	13.76	0.59	.444
Copulation latency df = 90 *R* ^2^ = .32	SSB treatment	209.47	93.00	5.07	.024
	Competition treatment	−31.24	89.19	0.12	.726
	SSB × Competition	325.29	130.31	6.23	.012

*Note*: GLMs and ANOVA results were shown for the number of copulations (male–female mounts), the mean duration of mounts, and copulation latency (time to first copulation). Model terms include the effect of SSB treatment (“control” as reference) on mating behavior, competition treatment (“without competition” as reference) on mating behavior and their interaction.

### 3.2 No effect of same‐sex behavior on reproductive success

**FIGURE 3:**
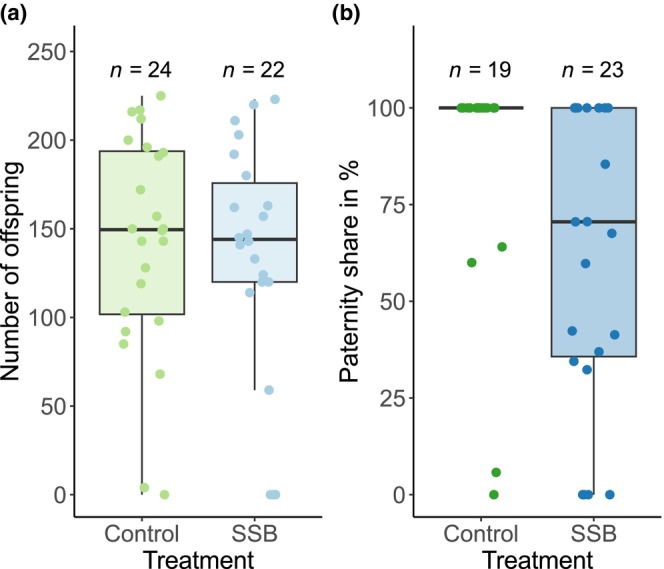
(a) Comparison of reproductive success between control males (green) and males subjected to same‐sex mating trials (blue) of males in mating trials with a single female mating partner without competition. (b) Comparison of paternity share between control males (green) and males subjected to same‐sex mating trials (blue) in mating trials with a single female mating partner and facing an Rd competitor.

In the next step, we tested for the effect of the SSB treatment on reproductive success. First, we used total offspring numbers in an assay where males faced no competition in mating trials. There was no significant difference in the number of offspring between SSB‐treated and control males (Figure 3a, Table 2). Second, we compared the paternity share between the treatments. Here, the focal males faced an Rd competitor in the male–female mating trials. There was a non‐significant trend towards a lower paternity share for males who were exposed to SSB compared to control males (Figure 3b, Table 2).

**TABLE 2:** The effect of exposure to same‐sex behavior on the total offspring number of males (treatment without competition) and paternity share of males (treatment with competition).

Treatment	Trait	df	*R* ^2^	*F*	*p*
Without competition	Offspring number	1, 46	.01	0.51	.480
With competitor	Paternity share	1, 42	.09	3.82	.057

### 3.3 Intensity of same‐sex mating behavior did not predict future reproductive success

**FIGURE 4:**
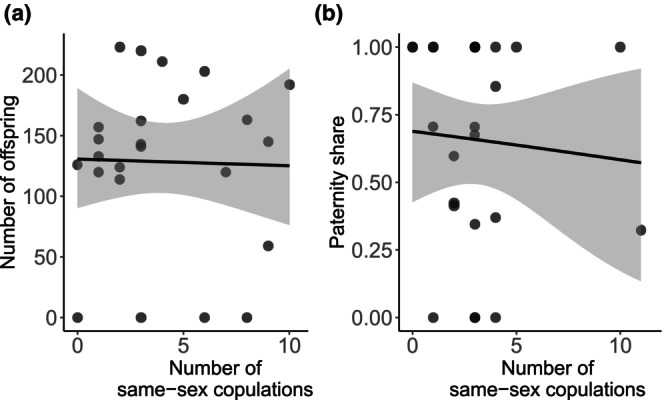
Relationship between the (a) number of offspring or (b) paternity share of the focal and the number of same‐sex copulations obtained from SSB‐treated males after male–female trials (a) without competition or (b) with the competition. Lines represent the fit of the GLM (Table 3) with gray areas as 95% CI.

In addition, we explored if the actual frequency of SSB of males subjected to the SSB treatment predicted future reproductive success. The number of same‐sex copulations did not predict offspring number in males facing no competitor (Figure 4a) or paternity share of males facing a competitor (Figure 4b, Table 3).

**TABLE 3:** Relationship between the number of offspring by SSB‐treated males and the number of same‐sex copulations for mating trials without competition. For mating trials with competition, the relationship between paternity share and the number of same‐sex copulations is shown.

Treatment	Trait	df	Estimate	SE	*t*	*p*
Without competition	Offspring number	22	−0.05	0.05	−1.01	.323
With competition	Paternity share	23	−0.05	0.13	−0.34	.737

### 3.4 No cost of same‐sex copulations during different‐sex mating trials

In the different‐sex mating trials including competition, some of the focal males performed SSB mounting the Rd competitor (25 of 54 cases). We therefore tested if engaging in SSB reduced the number of offspring produced. There was no significant relationship between the number of same‐sex copulations and the number of offspring in SSB‐treated or control males (Table 4). Overall, control males performed significantly more SSB during the different‐sex mating trials compared to males exposed to SSB in the same‐sex mating trials (two‐tailed Student's *t*‐test: df = 35.60; *t* = −2.99; *p* = .005).

**TABLE 4:** Relationship between the number of offspring and the number of same‐sex copulations obtained from SSB‐treated males or control males in male–female trials including an Rd competitor.

Model term	Estimate	SE	*χ* ^2^	*p*
Number of same‐sex copulations	−0.01	0.14	0.07	.796
SSB treatment	0.02	0.30	0.29	.589
Number of same‐sex copulations × SSB treatment	0.24	0.32	0.58	.446

*Note*: GLMs (df = 50) and ANOVA results shown for the effect of SSB number on offspring number, SSB treatment (“control” as reference) on offspring number, and their interaction.

### 3.5 No indirect sperm translocation through same‐sex copulations

Finally, we tested for a possible benefit of SSB by means of indirect sperm translocation. An indirect transfer of sperm from one male to a female may occur through an intermediate second male. If it occurred in the present experiment, part of the offspring of females in the different‐sex mating trials without Rd competitors should have expressed the dominant Rd marker, indicating sperm translocation from Rd males in the same‐sex mating trials. In 25 out of 27 cases, the focal male copulated with a Rd male during the same‐sex mating trials and in 14 cases the focal was mounted by a male Rd competitor. Overall, in all but one of the 27 same‐sex trials the focal was either mounted or mounting. No Rd offspring were subsequently observed, suggesting the absence of indirect sperm translocation. A previous study by LeVan et al. (2009) reported six instances of indirect sperm translocation out of 172 cases, which is not significantly different from the present data (*χ* ^2^ = 0.14; *p* = .704).

## 4 DISCUSSION

Same‐sex sexual behavior (SSB) is often viewed as an evolutionary paradox, even though it occurs in diverse animal groups (Bailey & Zuk, 2009; Monk et al., 2019; Scharf & Martin, 2013). In the present study, we found no evidence that SSB has fitness effects. Males exposed to the SSB treatment prior to mating with a female did not have significantly less offspring compared to control males, indicating that their ability to fertilize females was not impaired by SSB. Moreover, we found that SSB exposure did not impair the reproductive competitiveness of males suggesting that SSB does not lead to significant sperm depletion. With regards to the possible benefits of SSB, we found no evidence for indirect sperm translocation via an intermediary male.

### 4.1 Limited evidence that same‐sex matings alter subsequent mating behavior

We hypothesized that SSB might affect the subsequent mating behavior of males when mating with a female. Our data show that males subjected to SSB did not differ in copulation number or duration from control males. Nevertheless, males that engaged in SSB started copulating with a female significantly later than control males. This suggests that SSB had little influence on the mating behavior of males. Still, the increase in copulation latency might suggest a slight effect of sperm limitation or physical exhaustion, as males who engaged in SSB seemed to delay copulating with the females. Overall, our data provide limited evidence that SSB modulates the subsequent mating behavior of *T. castaneum* males.

### 4.2 No evidence for fitness effects of same‐sex behavior

Relatively little is known about the costs of SSB (Bailey & Zuk, 2009; Dukas, 2010; Maklakov & Bonduriansky, 2009; Monk et al., 2019). In *T. castaneum*, males kept in same‐sex groups did not suffer lower insemination success or last‐male paternity share 24 h after exposure to SSB (LeVan et al., 2009). However, it remained unclear how SSB affects the reproductive success and reproductive competitiveness of males recently exposed to SSB. We investigated how variation in same‐sex copulations impacted male reproductive success in the absence of male–male competition over fertilization and found that males participating in the same‐sex mating trials did not differ in their reproductive success compared to control males. This suggests that same‐sex copulations do not significantly influence subsequent reproductive success in *T. castaneum*.

In addition to male reproductive success in the absence of competition, we also estimated male reproductive competitiveness in an assay that allowed for competition over fertilizing the female. Males that engaged in same‐sex sexual encounters only showed a non‐significant trend towards a reduced paternity share compared to control males. This suggests that recent same‐sex copulations do not have a strong, negative impact on the reproductive competitiveness of *T. castaneum* males in subsequent copulations with females. Nevertheless, prolonged phases of SSB might still have a negative impact on male reproductive success along with pre‐ and post‐copulatory episodes of sexual selection (Spratt, 1980). In addition, our experiment did not include the fitness costs of SSB imposed by the time used for this behavior. Nevertheless, SSB with the Rd competitor male during the male–female mating trials did not influence the reproductive success of males, suggesting little or no time costs. In addition, it seems plausible that mechanisms of sex recognition to prevent SSB would also involve a time cost.

One hypothesis that would explain a potential negative impact of SSB on competitive reproductive success is that SSB could lead to sperm limitation in subsequent matings (LeVan et al., 2009; Sales et al., 2018). The non‐significant trend for a reduction in paternity share when males had performed SSB might suggest that sperm limitation could play a minor role in the costs of SSB in *T. castaneum*. Nevertheless, there was no correlation between the offspring number of SSB‐treated males and the number of same‐sex copulations performed in the same‐sex mating trials. An alternative reason for a lower paternity share in sperm‐limited males is reduced mating success. This would be in line with previous observations that *T. castaneum* males with low sperm quantity did not engage in sexual behavior (Bloch Qazi et al., 1996). Yet, our data suggest that exposure to SSB did not lead to behavioral changes in mating frequency, which would have indicated an effect of sperm limitation on mating success. Still, the increase in copulation latency in SSB‐treated males suggests that sperm limitation through SSB had an effect in delaying future copulations. More direct tests for the possible effect of SSB on sperm limitation are needed, for example, by measuring sperm quantity in males after performing SSB.

A limitation of all studies using experimental approaches manipulating the exposure to SSB is that also the social experience of males is altered. Importantly, when exposing males to SSB group size is manipulated as well. We minimized the influence of group size, by exposing the focal males only to two potential same‐sex partners for a limited time of 40 min. Hence, we do not expect, but cannot exclude, that the differences in social experience other than SSB affected treated males compared to control males.

### 4.3 Costs and benefits of same‐sex mating behavior

A constraint of the present study is that it only addressed the net effect of SSB and might have failed to identify potential costs and benefits. For example, the potential benefits of SSB might have been masked by costs in the present data. In addition, the costs of SSB are likely influenced by the ecological conditions, for instance, food stress might render SSB more costly. Nevertheless, there was also no conclusive evidence for any benefits of SSB in past studies (LeVan et al., 2009; Sales et al., 2018). LeVan et al. (2009) proposed that SSB in *T. castaneum* may function to dispose of old, low‐quality sperm. If SSB exists as a means for discarding old, low‐quality sperm to produce new, good‐quality sperm (LeVan et al., 2009), males that performed SSB should have had more offspring than control males. Our data suggest that SSB‐treated males did not gain fitness by disposing of old sperm. In the present experiment, males were approximately for 10–20 days fully adult, hence there might have been limited potential for sperm aging. Nevertheless, the typically high‐population density and high‐mating rate in this species (Wool, 1967) suggest that males usually do not experience long periods of sperm aging. *T. castaneum* is known to have a higher copulation frequency after isolation periods (Taylor & Sokoloff, 1971; Wool, 1967), but the higher number of copulations by isolated males is likely a result of an increased drive to mate, not an increased drive to engage in SSB to discard old sperm.

There are also theoretical reasons that render the sperm disposal hypothesis unlikely to apply in *T. castaneum*. Sperm dumping is unlikely to be beneficial to males since even old, low‐quality sperm could be used to fertilize a female. Hence, whenever a female is available to mate with, males should prefer different‐sex matings over SSB. In *T. castaneum*, the high‐population densities make it likely that males have continuous access to females under natural circumstances. Furthermore, *T. castaneum* females can use stored sperm for up to 140 days (Bloch Qazi et al., 1996), and control males in this study showed no effect of isolation on their ability to fertilize females. Hence, it is questionable that sperm aging would have a strong effect on fertilization success in this species – at least in the absence of sperm competition. However, sperm dumping may play an important role in other species, such as crickets (Kumashiro et al., 2003; Reinhardt & Siva‐Jothy, 2005), where males autonomously expel sperm.

### 4.4 Do *T. castaneum* males mate indiscriminately?

Overall, our results support previous findings suggesting that *T. castaneum* males mate indiscriminately with males and females, with low or no fitness costs associated with SSB. Nevertheless, we found that males from the SSB treatment performed less SSB with the Rd competitor compared to control males in the different‐sex mating trials. One explanation might be that the increased copulation latency of SSB‐treated males reduced the opportunity for SSB. Alternatively, males might use their experience of SSB to adjust their future behavior towards mating with a female. This would be in line with past findings in *Drosophila* adjusting SSB according to social experience (Bailey et al., 2013; Svetec & Ferveur, 2005). Still, the absence of evidence for the costs of SSB, past studies on the frequency of SSB (Sales et al., 2018; Serrano et al., 1991, 2000; Taylor & Sokoloff, 1971), as well as theory (Lerch & Servedio, 2021; Monk et al., 2019; Richardson & Zuk, 2023), suggest that the mating of male *T. castaneum* resembles more closely sexual indiscrimination.

### 4.5 No occurrence of indirect sperm translocation

We found no evidence that indirect sperm translocation via an intermediate male occurs in *T. castaneum*. There were no Rd offspring in any of the trials without competition. LeVan et al. (2009) reported only six instances of indirect sperm translocation out of 172 cases (each only 0.5% of the female's total progeny) and this result was not significantly different from the present data. Previous studies on indirect sperm translocation via an intermediate female have found widely contrasting results, with indirect sperm transfer being common in one study (Haubruge et al., 1999), but non‐existent in a replication of the experiment using a different paternity marker (Tigreros et al., 2009). More data is needed on indirect sperm translocation via both males and females to determine whether it occurs only in certain strains of *T. castaneum*. Since the sample size in our study was smaller than that of LeVan et al. (2009) and indirect sperm transfer occurred in only 3.45% of their cases the results do not necessarily contradict each other. They do suggest, however, that indirect sperm translocation via an intermediate male is not common in *T. castaneum* and is hence highly unlikely to convey a sufficiently significant adaptive advantage to explain the origin or maintenance of SSB in this species.

## 5 CONCLUSIONS

Taken together, our data indicate that *T. castaneum* males faced no detectable fitness consequences of performing same‐sex sexual behavior prior to mating with a female. *T. castaneum* beetles copulate frequently (Wool, 1967) and are highly polygamous (Lewis & Iannini, 1995; Martin et al., 2015), so that costs of same‐sex mating may be smaller than the benefits of copulating frequently and indiscriminately (Lerch & Servedio, 2021; Monk et al., 2019; Scharf & Martin, 2013). There is growing theoretical and experimental evidence suggesting that same‐sex sexual behavior entails lower costs than sex discrimination (Bailey & French, 2012; Engel et al., 2015; Hoving et al., 2011; Lerch & Servedio, 2021; Richardson & Zuk, 2023). This also seems to be the case in *T. castaneum* (Sales et al., 2018; Serrano et al., 1991, 2000; Taylor & Sokoloff, 1971) and our data corroborate this view by providing evidence that fitness consequences, if any, are small.

## AUTHOR CONTRIBUTIONS

**Birte M. Martens:** Conceptualization (supporting); data curation (supporting); formal analysis (supporting); investigation (lead); methodology (supporting); writing – review and editing (supporting). **Oliver Y. Martin:** Conceptualization (supporting); methodology (supporting); writing – review and editing (equal). **Tim Janicke:** Conceptualization (supporting); formal analysis (supporting); funding acquisition (lead); methodology (equal); project administration (supporting); supervision (equal); visualization (supporting); writing – review and editing (equal). **Lennart Winkler:** Conceptualization (lead); data curation (lead); formal analysis (lead); investigation (equal); methodology (equal); project administration (equal); supervision (supporting); visualization (lead); writing – original draft (lead); writing – review and editing (lead).

## CONFLICT OF INTEREST STATEMENT

The authors have no competing interests to declare.

## Data Availability

Analyses reported in this article can be reproduced using the published data and code (Martens et al., 2024; https://doi.org/10.5281/zenodo.10254987).

## APPENDIX 1

### ANALYSES USING AN ALTERNATIVE 39‐S THRESHOLD FOR MATING INTERACTIONS

The following tables replicate the analyses of the main manuscript using an alternative threshold for successful matings of 39 s instead of 29 s.

#### Male–female mating behavior

**TABLE A1:** Effect of same‐sex sexual behavior treatment and competition treatment on the mating behavior of males during different‐sex mating trials.

Trait	Model term	Estimate	SE	*χ* ^2^	*p*
Number of copulations df = 88 *R* ^2^ = .11	SSB treatment	0.32	0.20	2.50	.114
	Competition treatment	−0.01	0.19	0.01	.954
	SSB × Competition	−0.70	0.30	5.62	.018
Mean duration df = 76 *R* ^2^ = .09	SSB treatment	−17.98	11.66	1.72	.190
	Competition treatment	−23.56	10.42	4.87	.027
	SSB × Competition	14.34	16.48	0.76	.384
Copulation latency df = 78 *R* ^2^ = .21	SSB treatment	175.79	88.30	3.96	.046
	Competition treatment	8.97	77.44	0.01	.908
	SSB × Competition	161.03	123.91	1.69	.194

*Note*: GLMs and ANOVA results were shown for the number of copulations (male–female mounts), the mean duration of mounts, and copulation latency (time to first copulation). Model terms include the effect of SSB treatment (“control” as reference) on mating behavior, competition treatment (“without competition” as reference) on mating behavior and their interaction.

#### Analyses of reproductive success

**TABLE A2:** ANOVA of the effect of exposure to same‐sex behavior on the total offspring number of males without competition.

Treatment	Trait	df	*R* ^2^	*F*	*p*
Without competition	Offspring number	1, 38	<.01	0.12	.721
With competitor	Paternity share	1, 37	.14	5.43	.025

#### The intensity of same‐sex mating behavior did not predict future reproductive success

**TABLE A3:** Relationship between the number of offspring by SSB‐treated males and the number of same‐sex copulations for mating trials without competition. For mating trials with competition, the relationship between paternity share and the number of same‐sex copulations is shown.

Treatment	Trait	df	Estimate	SE	*t*	*p*
Without competition	Offspring number	22	−0.05	0.05	−1.01	.323
With competition	Paternity share	23	−0.03	0.14	−0.21	.835

#### No cost of same‐sex copulations during different‐sex mating trials

**TABLE A4:** Relationship between the number of offspring and the number of same‐sex copulations obtained from SSB‐treated males or control males in male–female trials including an Rd competitor.

Model term	Estimate	SE	*χ* ^2^	*p*
Number of same‐sex copulations	−0.06	0.18	0.01	.930
SSB treatment	0.01	0.27	0.23	.629
Number of same‐sex copulations × SSB treatment	0.33	0.34	0.89	.345

*Note*: GLMs (df = 50) and ANOVA results shown the effect of SSB number on offspring number, SSB treatment (“control” as reference) on offspring number, and their interaction.
